# Comparison of ED95 of Butorphanol and Sufentanil for gastrointestinal endoscopy sedation: a randomized controlled trial

**DOI:** 10.1186/s12871-020-01027-5

**Published:** 2020-05-02

**Authors:** Xiaona Zhu, Limei Chen, Shuang Zheng, Linmin Pan

**Affiliations:** Department of Anesthesiology, the First Affiliated Hospital, Wenzhou Medical University, Shangcai village, Nanbaixiang town, Ouhai District, Wenzhou City, 325000 Zhejiang Province China

**Keywords:** Butorphanol, Sufentanil, Gastrointestinal endoscopy, Sedation

## Abstract

**Background:**

Butorphanol, a synthetic opioid partial agonist analgesic, has been widely used to control perioperative pain. However, the ideal dose and availability of butorphanol for gastrointestinal (GI) endoscopy are not well known. The aim of this study was to evaluated the 95% effective dose (ED_95_) of butorphanol and sufentanil in GI endoscopy and compared their clinical efficacy, especially regarding the recovery time.

**Methods:**

The study was divided into two parts. For the first part, voluntary patients who needed GI endoscopy anesthesia were recruited to measure the ED_95_ of butorphanol and sufentanil needed to achieve successful sedation before GI endoscopy using the sequential method (the Dixon up-and-down method). The second part was a double-blind, randomized study. Two hundred cases of painless GI endoscopy patients were randomly divided into two groups (*n* = 100), including group B (butorphanol at the ED_95_ dose) and group S (sufentanil at the ED_95_ dose). Propofol was infused intravenously as the sedative in both groups. The recovery time, visual analogue scale (VAS) score, hand grip strength, fatigue severity scores, incidence of nausea and vomiting, and incidence of dizziness were recorded.

**Results:**

The ED_95_ of butorphanol for painless GI endoscopy was 9.07 μg/kg (95% confidence interval: 7.81–19.66 μg/kg). The ED_95_ of sufentanil was 0.1 μg/kg (95% CI, 0.079–0.422 μg/kg). Both butorphanol and sufentanil provided a good analgesic effect for GI endoscopy. However, the recovery time for butorphanol was significantly shorter than that for sufentanil (*P* < 0.05, group B vs. group S:21.26 ± 7.70 vs. 24.03 ± 7.80 min).

**Conclusions:**

Butorphanol at 9.07 μg/kg was more effective than sufentanil for GI endoscopy sedation and notably reduced the recovery time.

**Trial registration:**

Chinese Clinical Trail Registry (Registration number # ChiCTR1900022780; Date of Registration on April 25rd, 2019).

## Background

The morbidity from gastric and intestinal cancer is ranked second and fifth highest for cancers in China, respectively [1]. Gastrointestinal (GI) examination has been used as a standard method for the diagnosis of esophageal, gastroduodenal, and colorectal disease. However, unbearable abdominal pain can be caused by the distension and traction of viscera during GI endoscopy, eventually resulting in poor conditions for observation and severe arrhythmia [2]. Presently, sedative drugs combined with analgesics are typically used to alleviate pain and nervousness during GI endoscopy.

Currently, opioid μ receptor agonists, such as sufentanil and fentanyl, are the most commonly used analgesics. The stomach and intestine are mainly innervated by the sympathetic and parasympathetic nervous systems [3] and the kappa receptor agonist is found at higher concentrations in the spinal cord thus is involved in relieving visceral pain [4]. Butorphanol is a kappa receptor agonist, which has the advantages of light respiratory depression, stable hemodynamics, a rapid onset, and a moderate effective duration [5], and it may be a more suitable intraoperative and postoperative analgesic for painless GI endoscopy.

Butorphanol is a more effective analgesic than morphine, while its respiratory depression is as low as 1/5 that of morphine [6]. At present, butorphanol can be safely applied as a maternal analgesic, especially for pregnant women with pre-eclampsia and chronic hypertension, it dose not cause severe fluctuations in blood pressure [7]. Butorphanol has also been used in outpatients undergoing laparoscopic tubal sterilization in the early stage [8], although the analgesic dose has not been standardized [9, 10]. It is imperative that the optimal butorphanol dose that produces analgesia and minimizes side effects during outpatient sedation is found.

The objective of this study was to detect the ED_50_, ED_95_, and 95% confidence intervals for butorphanol using the sequential method and to compared these to the ED_95_ dose of sufentanil to assess the feasibility and superiority of butorphanol in GI endoscopy.

## Methods

This clinical study was approved by the Hospital Ethics Committee of the First Affiliated Hospital of Wenzhou Medical University and was registered in the Clinical Trial Registration Center of China (ChiCTR1900022780). Informed consent was obtained from all individual participants included in the study. This study adhered to CONSORT guidelines.

This study was based on the medical records of ASA I-II patients aged 18 to 65 who underwent an outpatient GI endoscopy (diagnostic esophagogastroduodenoscopy and colonoscopy, without therapeutic procedures), who required anesthesia and an operation of no more than 30 min in duration at the endoscopy center from May to July 2019. Patients were excluded from the study based on the following criteria: not willing or able to finish the whole study; acute upper respiratory tract infection; hepatitis and renal failure; habitual sedative or analgesic use; analgesic use for acute pain; chronic fatigue syndrome; low potassium; myasthenia gravis; psychiatric disease; and allergy to butorphanol, sufentanil, or propofol.

This study was divided into two parts: (1) determination of the ED_95_ of butorphanol and sufentanil; (2) comparison of the clinical efficacy of butorphanol with the efficacy of the equivalent sufentanil.

### ED_95_ of butorphanol and sufentanil

All patients underwent routine GI preparation before endoscopy, fasting from solids for 8 h and liquids for 2 h before the operation. The anesthesia machine was inspected, and intravenous access was established. Before inducting anesthesia in the outpatient operating room, standard monitoring was applied, including for non-invasive blood pressure (BP), electrocardiogram (ECG), and oxygen saturation (SpO_2_), and the patients were placed in the left lateral position. All the patients received 3 L per minute supplemental oxygen via nasal inhalation and were asked to hold the facial mask themselves.

Butorphanol (Batch number: 190411BP, Jiangsu Hengrui Pharmaceutical Co., Ltd.) or sufentanil (Batch number: 3018511505, Yichang Humanwell Pharmaceutical Co., Ltd.) was slowly injected intravenously. Given the 3 min onset time, propofol (Batch number: 1811236 Beijing Fresenius Kabi Pharmaceutical Co., Ltd.) was administrated intravenously at a constant speed until the patient lost consciousness and dropped the hand-held mask, followed by a continuous intravenous infusion at a rate of 50–150 μg•kg^− 1^•min^− 1^.The bispectral index (BIS) was monitored (BIS Complete Monitoring System, Covidien), and a controlled BIS value of between 50 and 60 was maintained by adjusting propofol speed. Then, the endoscopy was begun (operated by the same gastroenterologist). If the patient showed “failed sedation” (definition of failed sedation: occurrence of gag reflex [11], coughing, or body movement during esophagogastroduodenoscopy, or body movement during colonoscopy) during the GI endoscopy, an additional propofol dose of 0.5–1 mg/kg was administered. Once the SpO_2_ fell to 90%, assisted ventilation with oxygen via a facial mask was applied. If the heart rate dropped below 45 beats per minute, atropine (0.5 mg) was applied. If the mean arterial pressure was less than 50 mmHg, ephedrine 5–10 mg was administered. After surgery, the patients were transported to the postanesthesia care unit (PACU) to rest and recover.

### Dixon up-and-down method

The dose of butorphanol administered to each patient was determined by the Dixon up-and-down method [12]. According to geometric progression, the dose gradient was divided into six steps: 12.00, 10.00, 8.33, 6.94, 5.79, and 4.82 μg/kg. In a preliminary experiment, the ED_95_ of butorphanol for “successful sedation” (definition of successful sedation: without gag reflex, coughing, or body movement in esophagogastroduodenoscopy and body movement in colonoscopy) with propofol in outpatient GI endoscopy was 9.8 μg/kg. Therefore, the first patient was prescribed a dose of 10.00 μg/kg. The dose grade was increased or decreased using the up-down method based on the failure or success of the sedation in the previous patient. This process was repeated until there were nine cross-over pairs [13] (i.e., one successful sedation, followed by one failed sedation).

The dose of sufentanil given to each patient was also determined by the Dixon up-and-down method. According to geometric progression, the dose gradient was divided into six steps: 0.12, 0.1, 0.083, 0.069, 0.058, and 0.048 μg/kg. In the preliminary experiment, the ED_95_ of sufentanil for “successful sedation” with propofol in outpatient GI endoscopy was 0.085 μg/kg. Thus, the first patient was prescribed a dose of 0.083 μg/kg. The following process was similar to that used for testing the ED_95_ of butorphanol.

### Comparison with sufentanil

#### Groups

Two hundred cases of painless GI endoscopy patients were recruited. The patients were randomly divided into two groups: the butorphanol group (group B, *n* = 100) and the sufentanil group (group S, n = 100).

#### Anesthesia methods

This part of the study was double-blind and randomized. The patients were grouped according to the envelope method. The dispensing nurse dispensed the drugs according to the directions of the anesthetist. The preoperative preparation and anesthesia methods were the same as in the first part of the study and were performed by the anesthetist. The ED_95_ dose of butorphanol (9.07 μg/kg) was administered to group B. The ED_95_ doses of sufentanil (0.1 μg/kg) was administered to group S. The ED_95_ doses of butorphanol and sufentanil were estimated in the first part of the study. Postoperative indications in the PACU were evaluated and recorded by another postoperative observer who was blinded to the group division.

#### Efficacy measurements and variables

The primary outcome in this study was the recovery time, which represented the time from completion of the examination and to the patient’s departure from the PACU. The standards for hospital discharge were our outpatient operational standards [14] (including vital signs, pain, orientation, dizziness, and walking). The secondary outcomes included the demographic and medical data, i.e., the incidence of respiratory depression (respiratory rate < 10 beats/min or SpO_2_ < 90% in nasal catheter oxygenation with 3 L/min), the incidence of circulatory inhibition (MAP < 50 mmHg or HR < 45 beats /min), dosage of propofol, the incidence of failed sedation, fatigue severity scores (assessed with an 11-point (0–10) scale [15] 15 min after awakening time), VAS score of abdominal pain (15 min after awakening time), value of hand grip strength before and 15 min after operation (assessed using an electronic hand dynamometer [EH101, Camry Co. Zhongshan, China]), the incidence of nausea and vomiting, and dizziness after awakening.

### Statistical analysis

SPSS statistical software (IBM Corporation, version 19) was used for statistical analyses. The median effective dose (ED_50_), ED_95_, and the 95% confidence intervals (CI) of butorphanol and sufentanil were determined by binary regression (probit) [16].

The sample size in part two was evaluated by PASS 11.0. The primary indicator was recovery time. The pre-experimental measurements showed that the recovery time was 22.12 ± 7.9 min in the butorphanol group and 25.57 ± 8.1 min in the sufentanil group. A sample size of 93 in each group was determined to be required for a β value of 0.10 and an α value of 0.05. Considering the loss of data and the number of patients who could not be interviewed after endoscopy, 100 patients were selected in each group to ensure that the experiment had a large enough sample size.

Normally distributed data were analyzed with the mean ± standard deviation, and a two independent sample *t*-test was used to evaluate the differences between the two groups. The non-parametric data were analysed using the median (Q1, Q3) or ratio, and a non-parametric test was used to evaluate the differences between the two groups. The complication rates were compared using a four-square table Chi-squared test. A *P*-value < 0.05 was considered to be statistically significant.

## Results

**Fig. 1:**
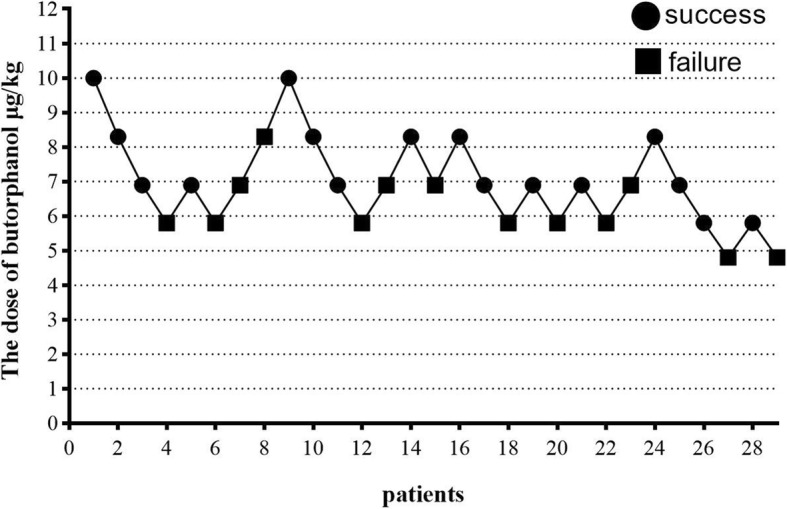
Responses (successful sedation) of 29 consecutive patients who received butorphanol as an analgesic during GI endoscopy

**Fig. 2:**
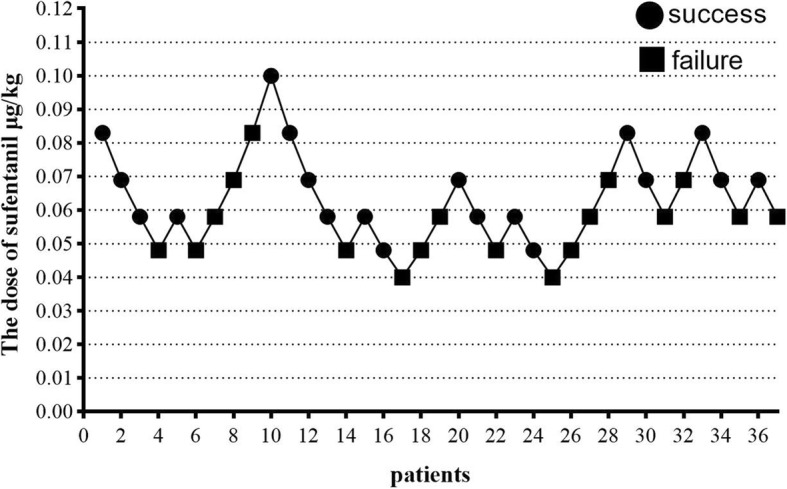
Responses (successful sedation) of 37 consecutive patients who received sufentanil as an analgesic during GI endoscopy

The data of 30 patients were screened in the first part of the study. One patient was excluded due to poor GI preparation, thus 29 cases remained. The individual responses to butorphanol assessed using Dixon’s up-and-down method are shown in Fig. 1. The ED_50_ of butorphanol for inhibiting body movement during painless GI endoscopy was 6.58 μg/kg (95% CI, 5.57–7.49 μg/kg), and the ED_95_ of butorphanol was 9.07 μg/kg (95% CI, 7.81–19.66 μg/kg) for the same procedure. A total of 37 patients were included in the second part of the study. The individual responses to sufentanil assessed using Dixon’s up-and-down method are shown in Fig. 2. The ED_50_ of sufentanil for inhibiting body movement during painless GI endoscopy was 0.060 μg/kg (95% CI, 0.048–0.073 μg/kg) and the ED_95_ of sufentanil was 0.100 μg/kg (95% CI, 0.079–0.422 μg/kg) for the same procedure. No significant circulatory or respiratory depression occurred during the operation.

A total of 200 patients were recruited to completed the second part of the study, and their data were analyzed to produce the final results (*n* = 100 per group). The characteristics of the enrolled subjects are shown in Table 1. There were no significant differences between the two groups regarding patient age-gender composition, SBP (Systolic Blood Pressure), heart rate, weight, height, BMI, GI endoscopy operation time, preoperative hand grip strength, and ASA (American Society of Anesthesiologists) grade composition (*P* > 0.05).

**Table 1:** General comparison between group S and group B

	S group(*n* = 100)	B group(*n* = 100)
Weight, kg	63 ± 11	64 ± 10
Sex (male, female)	(60, 40)	(63, 37)
SBP, mmHg	128 ± 15	130 ± 21
Heat rate, beats/min	69 ± 17	77 ± 13
Height, cm	165 ± 8	166 ± 8
BMI, kg/m^2^	23.3 ± 3.0	23.4 ± 2.8
Operation time, min	14.4 ± 4.9	14.6 ± 4.9
Preoperative hand grip strength, kg	42.9 ± 9.5	44.4 ± 8.9
ASA classification, I/II	60/40	66/34

*ASA* American Society of Anesthesiologists ASA physical status classification. Normally distributed statistics dates were mean ± SD, and a two independent sample t-test was used to evaluate the differences between the two groups. Sex and ASA classification were ratio and were compared by χ2 test. There were no significant differences between the two groups (*P* > 0.05)

There were no statistically significant differences in the incidences of respiratory depression (*P* = 0.469), circulatory inhibition (*P* = 0.489), failed sedation (*P* = 0.352), dizziness (*P* = 0.205), and propofol dosage (*P* = 0.171). Compared to group S, group B showed lower fatigue severity scores (*P* = 0.001) and better postoperative hand grip strength (*P* < 0.001). Furthermore, the recovery time for group B was significantly shorter than for group S (*P* = 0.012). The incidence of nausea and vomiting for group B was significantly lower than for group S (*P* = 0.014), as shown in Table 2.

**Table 2:** Comparison of the indicators between group S and group B

	S group(n = 100)	B group(*n* = 100)	*P* value
Incidence of respiratory depression	11%	8%	0.469
Incidence of circulatory inhibition	12%	9%	0.489
Dosage of propofol, mg	222.6 ± 38.4	215.0 ± 39.7	0.171
Incidence of failed sedation	7%	4%	0.352
VAS score	2 (1,3)	2 (1,2)	0.001*
Fatigue severity scores	2.18 ± 1.30	1.66 ± 0.87	0.001*
Postoperative grip strength, kg	31.8 ± 6.8	35.5 ± 7.7	0.000*
Incidence of nausea and vomiting	7%	0	0.014*
Incidence of dizzness	6%	11%	0.205
Recovery time, min	24.03 ± 7.80	21.26 ± 7.70	0.012*

The VAS scores are the median (Q1, Q3). The Mann-Whitney U-test was used to evaluate the differences. Normally distributed statistics dates were mean ± SD, and a two independent sample t-test was used to evaluate the differences. Ratios were compared by χ2 test.* *P* < 0.05

## Discussion

In our study, the ED_50_ of butorphanol for inhibiting body movement in painless GI endoscopy was 6.58 μg/kg, (95%CI: 5.57–7.49 μg/kg) and the ED_95_ was 9.07 μg/kg (95%CI: 7.81–19.66 μg/kg). The ED_50_ for inhibiting body movement of sufentanil in painless GI endoscopy was 0.060 μg/kg (95% CI, 0.048–0.073 μg/kg) and the ED_95_ was 0.100 μg/kg (95% CI, 0.079–0.422 μg/kg). In the second part of our study, the primary indicator (recovery time) in group B was significantly shorter than that in group S. Compared to group S, the VAS score, fatigue severity score, incidence of postoperative nausea and vomiting were lower in group B.

A sequential method was used to accurately select the optimal doses of butorphanol and sufentanil for GI endoscopy. An advantage of this method is that it can be used to evaluate the efficacy of drugs using fewer cases over a short time. The ED_95_ values of butorphanol and sufentanil were 9.07 μg/kg and 0.1 μg/kg, respectively, which were close to the doses used in the first patients in whom we administered the drugs (10 and 0.83 μg/kg, respectively). In our study, we confirmed that there was no difference in the incidences of successful sedation using the ED_95_ of butorphanol and sufentanil during GI endoscopy.

With a published in vitro affinity for opioid receptors of 1:4:25 (mu: delta: kappa), butorphanol has been known to act on kappa-opioid receptors of the upper spinal cord to inhibit nociceptive stimulus conduction [5]. Ozaki et al. demonstrated that kappa-, but not mu- or delta-, opioid receptor agonists modulate visceral sensations conveyed by the vagal afferent fibers innervating the stomach [17]. Soichiro et al. reported that butorphanol-induced visceral chemical antinociception was entirely blocked by pretreatment with a kappa-opioid receptor antagonist [18]. Kappa receptor shows absent related to respiratory depression, nausea, and vomiting. The mu receptor has strong effects on respiratory depression and is associated with nausea and vomiting [19]. Our experimental results are consistent with previous findings; they also confirm that butorphanol is less likely to cause nausea and vomiting and show that butorphanol resulted in a lower postoperative VAS score than the pure mu-opioid receptor agonist sufentanil at the ED_95_ dose. The most likely reason for this is the difference between the kappa and mu receptors. In addition, the doses of butorphanol and sufentanil used in our study were low, thus led to a low incidence of respiratory depression and did not result in a significant difference between them. The duration of the analgesic effect of butorphanol is about 4 h. Although the average examination time of painless GI endoscopy is not that long, the patient still needs excellent analgesia after waking up. PremyslFalt et al. reported that, with an intravenous injection of 2 mg midazolam after routine air-inflated GI endoscopy, 1% of patients still reported abdominal pain and 2% of patients had flatulence during the 3 h and 30 min after the procedure had finished [20]. It is essential to have excellent analgesia during this period, and butorphanol is a suitable choice.

Postoperative fatigue influences the emotional and mental state of the patients after surgery and affects their recovery [21]. Sufentanil is the classic analgesic drug used for painless GI endoscopy. However, during its clinical in our study, several patients experienced fatigue phenomenon lasting more than 1 h. In C^11^- labeled positron emission tomography, it was found that exercise can evoke and be related to changes in μ receptors in most of the limbic system, and deactivation of the μ receptor is the main reason for fatigue [22]. There was a strong correlation between grip strength and fatigue, after adjustment for age and height, that was independent of physical activity levels [23, 24]. Butorphanol resulted in less fatigue than sufentanil according to both subjective and objective indicators. We speculated that butorphanol can reduce visceral pain in GI endoscopy, as it targets the kappa receptor and decreases deactivation of the μ receptor, thereby reducing postoperative fatigue.

Compared with sufentanil, we believe that butorphanol, reduces postoperative nausea and vomiting, improves postoperative analgesia, and reduces postoperative fatigue, thus reducing the time at PACU after GI endoscopy.

This study had several limitations: (1) Fatigue is a multi-factor subjective experience. We only evaluated one objective indicator of fatigue, grip strength, and used a simplified scale. (2) Clinical examinations of outpatients were usually incomplete. The existence of hidden diseases and different sensitivities to drugs in individuals may have affected the results of the trial.

## Conclusion

In summary, the ED_95_ for butorphanol in inhibiting body movement during painless GI endoscopy was 9.07 μg/kg. Butorphanol combined with propofol as anesthesia for GI endoscopy reduced the recovery time, and, therefore, presents an excellent sedation strategy.

## Data Availability

The datasets during and analyzed during the current study are available from the corresponding author on reasonable requests.

## Abbreviations

ED95: Effective dose
CI: Confidence intervals
GI: Gastrointestinal
ASA: American Society of Anesthesiologists
BP: Blood pressure
ECG: Electrocardiogram
SpO2: Oxygen saturation
BIS: Bispectral index
PACU: Postanesthesia care unit
